# Using high-density DNA methylation arrays to profile copy number alterations

**DOI:** 10.1186/gb-2014-15-2-r30

**Published:** 2014-02-03

**Authors:** Andrew Feber, Paul Guilhamon, Matthias Lechner, Tim Fenton, Gareth A Wilson, Christina Thirlwell, Tiffany J Morris, Adrienne M Flanagan, Andrew E Teschendorff, John D Kelly, Stephan Beck

**Affiliations:** 1 UCL Cancer Institute, University College London, 72 Huntley Street, London WC1E 6BT, UK; 2 Royal National Orthopaedic Hospital, Stanmore, Brockly Hill, Middlesex HA7 4LP, UK; 3 Division of Surgery and Interventional Science, UCL Medical School, University College London, London WC1E 6BT, UK

## Abstract

The integration of genomic and epigenomic data is an increasingly popular approach for studying the complex mechanisms driving cancer development. We have developed a method for evaluating both methylation and copy number from high-density DNA methylation arrays. Comparing copy number data from Infinium HumanMethylation450 BeadChips and SNP arrays, we demonstrate that Infinium arrays detect copy number alterations with the sensitivity of SNP platforms. These results show that high-density methylation arrays provide a robust and economic platform for detecting copy number and methylation changes in a single experiment. Our method is available in the ChAMP Bioconductor package: http://www.bioconductor.org/packages/2.13/bioc/html/ChAMP.html.

## Background

Copy number alterations (CNAs) have been implicated in the development and progression of many human malignancies, including prostate, bladder and breast cancer [1-4]. Since first described in the late 1990s, many platforms have been developed for assessing alterations in genomic copy number at an ever increasing resolution [5-9]. The latest version of copy number variation arrays can interrogate over one million loci, and have the ability to detect genomic alterations ranging from approximately 4 kb to over 2 Mb [10-13]; they are, however, limited in the size of small alterations detectable, due to the distance between loci interrogated (Table 1). As a result, many small/micro-deletions encompassing single genes may not be detectable [9].

**Table 1:** Genomic probe distribution

	**Affymetrix SNP 6.0**	**Illumina CytoSNP**	**Illumina 450 K methylation array**
Number of probes	945,806	296,715	485,577
Median intermarker distance (kb)	2.3	6.1	0.35
Mean intermarker distance (kb)	3.0	10.8	5.8

Number of probes, mean and median intermarker distance interrogating copy number alterations from Affymetrix SNP 6.0, Illumina CytoSNP and Infinium HumanMethylation450 BeadChip.

In parallel, arrays designed to interrogate epigenetic alterations, particularly DNA CpG methylation, have been developed. These arrays were initially designed based on immunoprecipitation (MeDIP) or enzymatic digestion followed by hybridization to a bacterial artificial chromosome or oligonucleotide CpG island array [14,15]. Subsequently, there has been a move towards arrays designed on the premise of SNP detection arrays, and applied to bisulfite converted DNA [16-18]. Probes are designed for the detection of C/T alterations based on the conversion of unmethylated cytosine with bisulfite. The relative ratio of methylated (C) to unmethylated (T) residues is then used to define the methylation state of a particular locus [16].

The integration of genomic and epigenomic data from the same sample is becoming increasingly popular as we try to garner a greater understanding of the complex mechanisms driving the development and progression of cancers. Although at present arrays still prove the most cost-effective method of assessing both copy number and DNA methylation state, this interest in integrating multiple data sets means a significant increase in costs associated with these projects. Huge international efforts are currently underway through the International Cancer Genome Consortium (ICGC) and the Cancer Genome Atlas (TCGA) projects to produce genomic and epigenomic data on a huge number of human cancers. At present these data are generated on separate array platforms, with over 6,200 SNP arrays and 6,300 methylation arrays used to date to generate genomic and epigenomic profiles from the same sample. This, therefore, not only doubles the cost but also the amount of specimen used. The latter is particularly important when considering the potential effects of tumor heterogeneity on disease development, where subtle areas of a tumor are genetically and epigenetically different, which may ultimately confer a different phenotypic trait, such as differing metastatic potential [19].

We therefore sought to assess if the Infinium HumanMethylation450 BeadChips (the methylation array of choice for the ICGC and TCGA) could be used to define regions of CNA as well as sites of aberrant CpG methylation. It has already been shown, for low density methylation arrays and high resolution whole genome bisulfite sequencing, that changes in genomic content do not impact on the ability of these arrays to accurately define the methylation state for individual loci and that these technologies also have potential utility in detecting CNAs [20-22]. As the Infinium methylation arrays are, in essence, SNP arrays, providing high density coverage of the genome, the question is do they have the sensitivity and specificity to detect CNAs with the same accuracy as existing technologies. This will not only allow analysis and ultimately the integration of both epigenetic and copy number from exactly the same DNA specimen, potentially important when considering the effects of tumor heterogeneity on disease development and progression [19,23], but will also significantly reduce the cost of integrated epigenomic cancer studies looking to incorporate both data types.

## Results and discussion

### Influence of copy number alteration on methylation state

**Figure 1:**
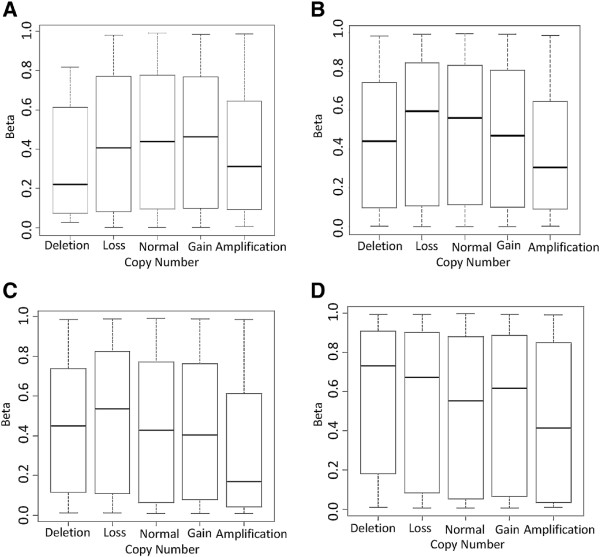
**Association of methylation state with copy number.** Box plots showing the influence of changing genomic content on methylation state (average beta value) inferred from SNP (CytoSNP and Affymetrix SNP6.0) and Infinium arrays, respectively, for **(A)** chondrosarcoma, **(B)** glioblastoma multiforme, **(C)** bladder cancer and **(D)** prostate cancer.

Prior to evaluating whether the Infinium array could detect CNAs, we first sought to assess whether alterations in genomic content influenced the methylation state inferred by the Infinium HumanMethylation450 BeadChips. Previous analysis of similar low density Infinium type arrays (GoldenGate) have shown that changes in DNA methylation are unaffected by copy number (CN) state [20]. Figure 1 shows the average beta value (methylation score) for all potential sites on the Infinium array as a function of CN determined from Affymetrix SNP6.0 or Illumina CytoSNP arrays from 11 chondrosarcoma and 74 glioblastoma multiforme (GBM) tumors. It also shows the average beta value for all potential sites on the Infinium array as a function of CN determined from an Affymetrix SNP6.0 array from 144 bladder cancer and 178 prostate cancer samples, respectively.

These data show that CN has little impact on methylation (Figure 1) in either series at regions of heterozygous loss or single copy gains when compared with regions of normal CN. However, there does appear to be an association when assessing homozygous deletion and amplification (*P* < 2.2e-16), where a significant negative correlation is observed with both data sets.

An association with beta value and homozygous loss was as expected as low/no signal does not allow accurate assessment of methylation; in fact, most probes in these regions fail to pass the Illumina signal quality detection *P*-value (defined by the comparison of signal from the target compared to that of negative controls (Illumina user manual)), and are removed in standard methylation analyses. Unexpectedly, however, a significant negative correlation was observed between regions of SNP array amplification and reduced beta values in all data sets. Unlike in regions of deletion, over 97% of probes in regions of amplification pass the detection *P*-value. On closer inspection, this negative correlation appears to be driven by the Infinium probe distribution. A higher proportion of probes in regions of focal amplification are located in CpG islands, which are predominately unmethylated, when compared with regions of normal ploidy [12,13,24]. Separating the Infinium probes within regions of amplification into CpG island-associated versus non-CpG island-associated confirmed this (Figure S1 in Additional file 1), with CpG island-associated probes having a mean beta of 0.28 compared with 0.62 for non-CpG island-associated probes (similar beta values are observed if regions of no change and gain are partitioned in a similar fashion). The inherent complex dynamics between CN and methylation means it is difficult to disentangle biology from systematic biases.

### Array artifact removal

Furthermore, as with other array-based platforms, technical artifacts, such as batch effects and genomic wave, may impinge on the accurate profiling of CNA form the Infinium arrays. A ‘genomic-wave’ artifact, a probe effect that correlates with surrounding genomic GC content and is commonly observed in other comparative genomic hybridization and SNP array platforms, and is also manifest on the Infinium arrays [25,26]. In order to help negate any effects of local CG content in calling CNAs, we performed a loess correction prior to CNA analysis, which estimates and removes the wave effects [25].

In a similar fashion, batch effects have been shown to have a substantial effect on high throughput array-based platforms, and are particularly apparent with the Infinium arrays, particularly when considering scale projects, such as the TCGA [27,28]. In order to help reduce variance attributed to batch as opposed to biological influence, we also incorporated batch effect removal with the ComBat function [29]. Batch effect removal significantly improved the correlation between replicate samples across differing batches (Figure S2 in Additional file 1): uncorrected R2 = 0.77 compared to batch-corrected R2 = 0.97. The correlation of replicate samples within a single array was R = 0.99, suggesting array position does not unduly affect signal intensity. All subsequent analysis where carried out on wave- and batch-corrected data (Figure S2 in Additional file 1).

It is well documented that the different Infinium assay designs (type I and type II) show considerable probe effects [16,30]. For example, when assessing methylation, the beta values derived from Infinium II probes were less accurate and reproducible than those obtained from Infinium I probes [30]; it has therefore been suggested (at least for methylation analysis) that the differing probe types be treated independently. We initially took this approach when utilizing these arrays to assess CN, as the intensities of the two probe types also show considerable differences [16,30].

### Copy number alteration profiling using Infinium methylation arrays

**Figure 2:**
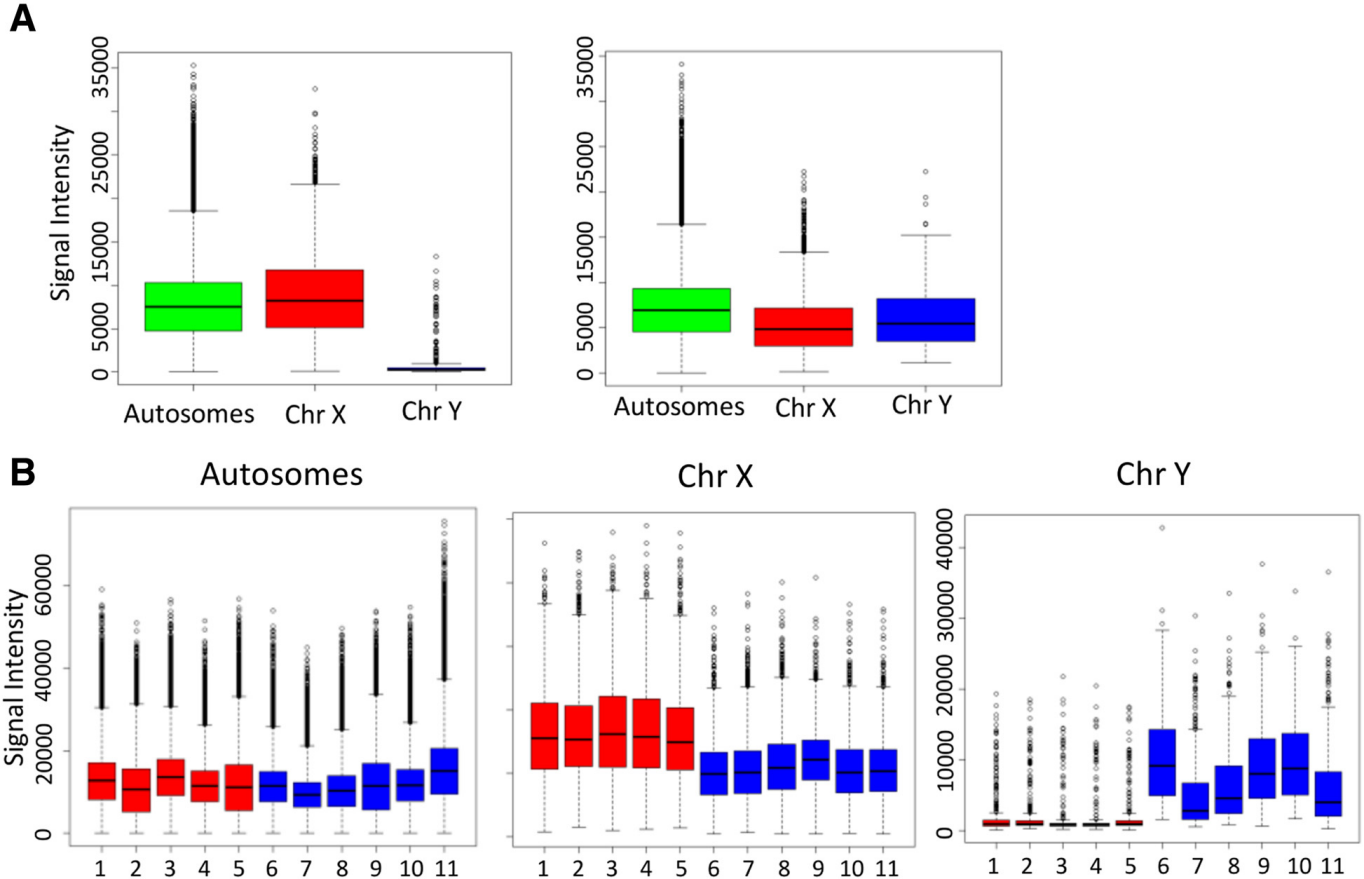
**Changes in Infinium probe intensities between sex chromosomes. (A)** Box plots of Infinium total signal intensities for autosomes and sex chromosomes for male and female patients (autosome (green) and sex chromosomes (chromosome X (red), chromosome Y (blue)). **(B)** Box plots of Infinium intensities for autosomes and sex chromosomes for 11 chondrosarcoma samples (females, red; males, blue).

Our initial motivation was to assess if the Infinium HumanMethylation450 BeadChips could provide information on genomic rearrangements with a level of accuracy comparable to current gold standard SNP arrays. As the Infinium arrays are, in essence, SNP arrays, with probes designed to interrogate the relative ratio of a methylated to unmethylated (C to T) template in bisulfite converted DNA, and as the methylation state (beta value) is defined by a relative ratio of methylated probe signal intensity to the total signal intensity of both methylated and unmethylated probes, it is logical to expect that these arrays may also allow assessment of CN. If total (unmethylated plus methylated) probe intensity is representative of CN, then the simplest of CN changes, that is, differences in the sex chromosomes between males and females, should be clearly detectable. Figure 2 shows the total signal intensities of the autosomal and sex chromosomes for normal reference DNA and 11 chondrosarcoma patients. These data clearly show a significant (*P* < 2.2e-16) difference between the autosomal chromosomes and sex chromosomes and that the Infinium methylation arrays can potentially detect single copy alterations.

**Figure 3:**
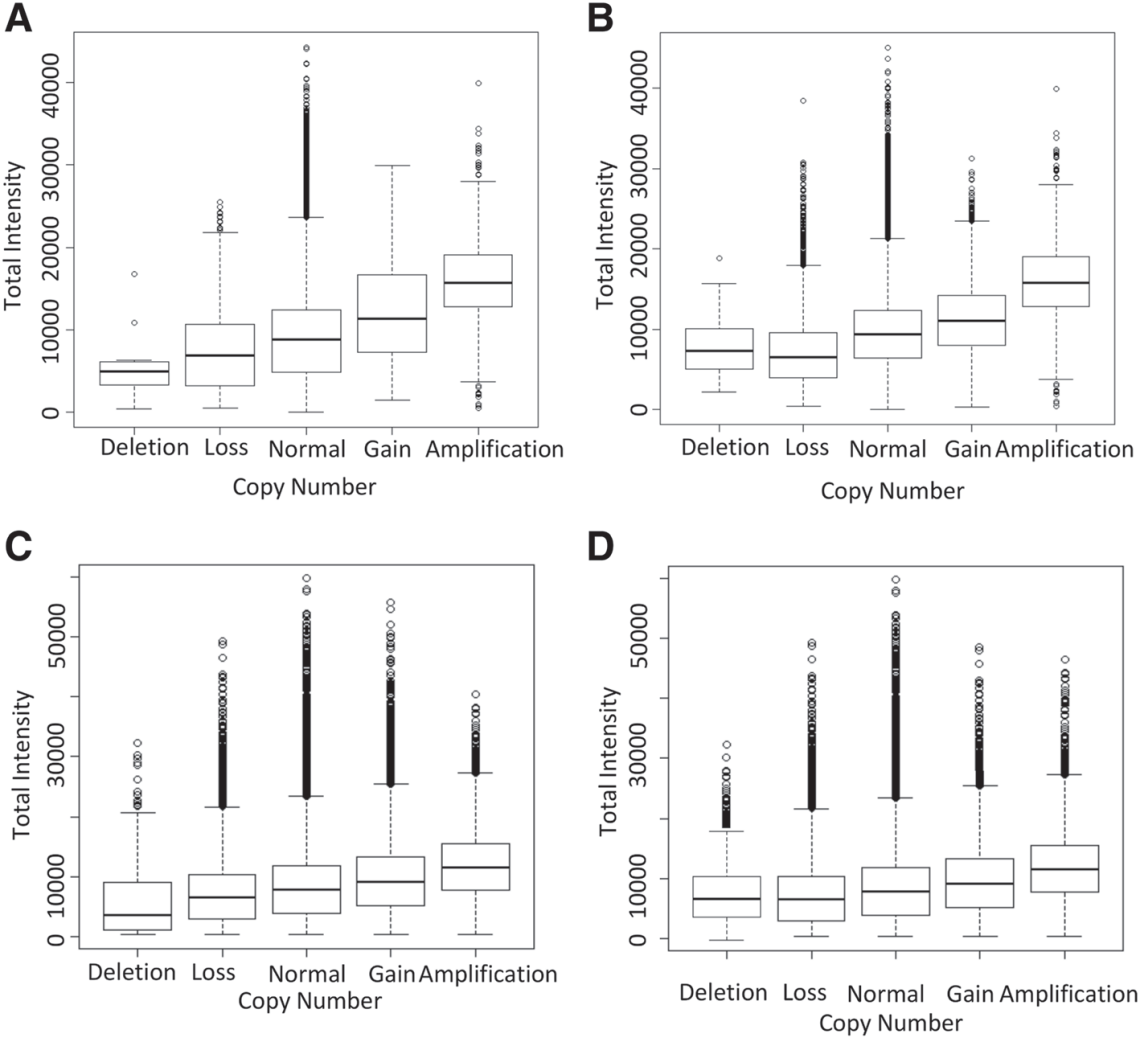
**Comparison of Infinium total probe intensity and changing copy number.** Box plots showing the association of total probe signal intensity from the Infinium arrays and copy number state inferred from SNP arrays for **(A)** chondrosarcoma, **(B)** glioblastoma multiforme, **(C)** bladder cancer and **(D)** prostate cancer.

We subsequently assessed the relationship between Infinium probe intensity and differing CNA states defined by SNP array from an in-house series of matched Infinium and CytoSNP arrays along with 386 samples from the TCGA project, representing three tumor types, GBM, prostate cancer and bladder cancer. As expected, regression coefficients confirmed that the mean Infinium signal intensity increases monotonically with CNA state (Figure 3), with a significant difference (*P* <0.0001) in mean Infinium signal at all levels of CN states, except for putative homo- and heterozygous loss in the GBM samples, where no difference is observed (*P* = 0.76). It should be noted that, for both sample cohorts, there were sufficient Infinium probes within regions of potential homozygous/heterozygous loss (defined on the SNP arrays (CytoSNP and SNP6.0)) to allow comparison, and while in theory no signal should be detectable when no copies exist (heterozygous loss), no two probes from the Infinium or SNP arrays overlap the same genomic loci, and that there is a stochastic component to both the assignment of CN and measurement of intensity that may account for this lack of correlation in regions of heterozygous/homozygous loss.

Finally, we sought to define CN profiles from Infinium array data. CNAs were identified using circular binary segmentation in the Bioconductor package DNAcopy [31]. We initially analyzed both probe types independently and evaluated the concordance of CNAs identified. Using the default parameters, type II probes appear to show a higher degree of ‘noise’ than the type I probes. Despite this, the concordance of CNAs called by both probe types (when considering large regions) is high (97%), although this is significantly lower when considering smaller focal alterations (24%). However, this may also somewhat reflect the differing genomic densities of the two probe types. Comparing overlapping regions only showed the CNA states generated from the two probe types to be highly correlated (R2 = 0.94, range 0.48 to 0.99; Figure S3 in Additional file 1), allowing the two probe types to be coalesced.

**Figure 4:**
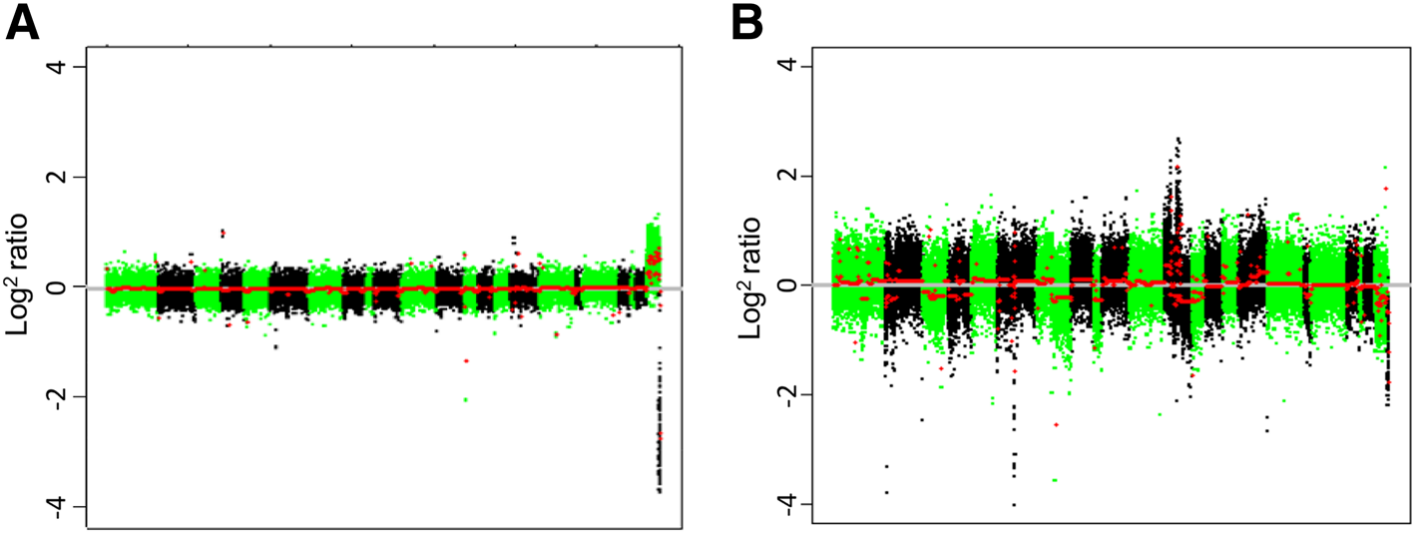
**Normal and malignant copy number profiles. (A)** CN profile for normal female versus male reference. **(B)** CN profile for a highly aneuploid cancer genome (versus male reference) derived from the Infinium arrays. Individual chromosomes are shown in green/black and segmented CN is shown in red.

To confirm that CNA analysis can detect single copy events, we compared normal reference DNA from single male and female subjects. Figure 4 shows example CNA profiles of reference male versus female samples, between which a significant difference in the CN state of the sex chromosomes is observed (*P* ≤ 0.0001), along with an example of a highly aneuploid malignant genome. These data indicate that the Infinium HumanMethylation450 BeadChips, when combined with circular binary segmentation, can detect both single copy and potentially high level CNAs.

### Correlation between Infinium and SNP array-defined CNAs

We next sought to assess whether the Infinium arrays could give a robust definition of CNAs compared to the gold standard SNP arrays for aneuploid malignant genomes. CNAs were determined from both SNP arrays as above, using the Bioconductor package DNAcopy for GBM samples. For bladder cancer and prostate cancer samples, processed CNA estimates were download directly from the TCGA project.

**Figure 5:**
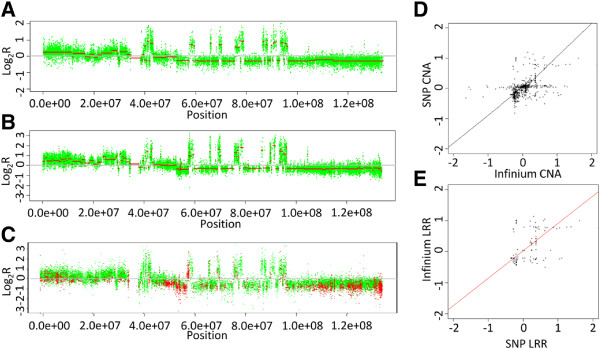
**Comparison of Infinium and SNP copy number profiles.** CN profiles for chromosome 12 for a single chondrosarcoma sample using **(A)** Infinium array, **(B)** CytoSNP and **(C)** combined Infinium and CytoSNP. **(D)** Scatter plot showing the correlation between Infinium CN and SNP array CN states from matched samples. **(E)** Scatter plot of Infinium and SNP array CNA states for chromosome 12 only.

We assessed the correlation of all 407 samples with paired Infinium and SNP array CNA profiles. In the majority of cases, global CN profiles from the different platforms appeared highly correlated with an average correlation coefficient of 0.91, ranging from 0.29 to 0.99, and show a similar frequency and amplitude of alterations. Figure 5 shows CN profiles from a single sample for chromosome 12 for both Illumina CytoSNP and Infinium 450Methylation Bead arrays as well as an overlay of these. It also shows the correlation between Infinium CNA and SNP array CNA profiles for the whole genome and for chromosome 12 (R2 = 0.96).

To assess the robustness of CNAs identified from the Infinium arrays, we compared them with CN profiles generated from a SNP array for matched samples. We initially assessed the agreement of large rearrangements (that is, alterations of >10 Mb) for both gains and losses. This analysis showed that a total of over 94% of large chromosomal gains and 97% of losses were identified by both Infinium and the SNP array, suggesting that the Infinium arrays show sufficient sensitivity to detect large scale, predominately single copy alterations.

### Copy number alteration detection sensitivity

**Figure 6:**
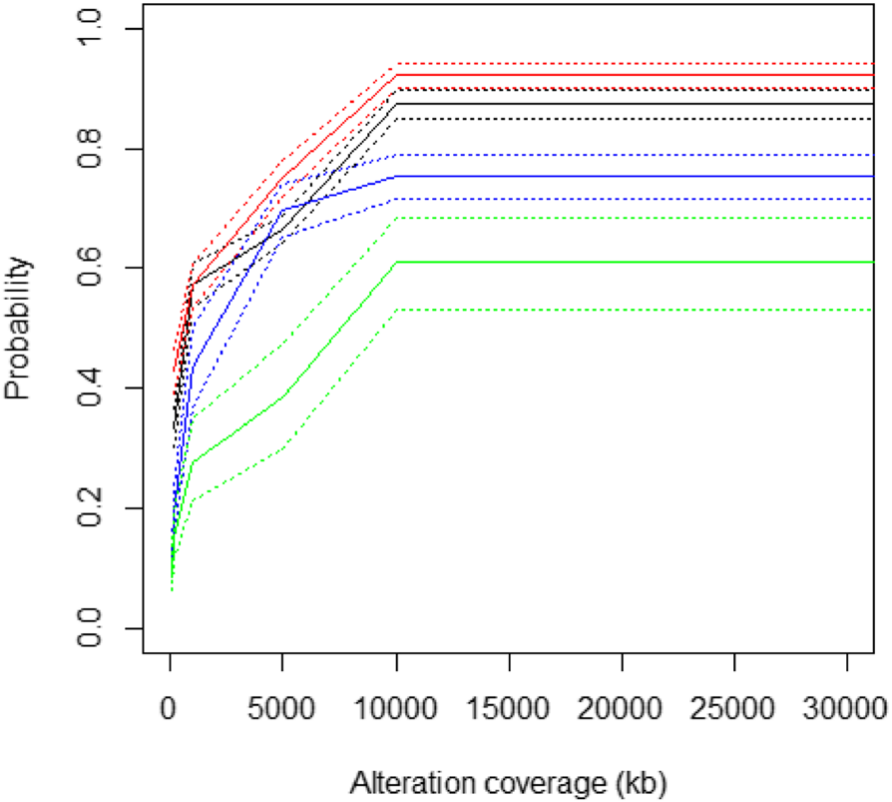
**Coverage plots of candidate true positives to assess the sensitivity and specificity of alterations defined by the Infinium arrays.** At each alteration threshold (deletion, blue; loss, red; gain, black; amplification, green) we counted the alterations that overlap between matched arrays. The number of overlapping regions (candidate true positives). Dotted lines represent 95% confidence intervals.

Besides the detection of large chromosomal rearrangements, we also sought to evaluate the ability of the Infinium arrays to detect focal alterations, including small (<1 Mb) high-level amplifications and homozygous deletions. We initially assessed the overlap between all regions of focal genomic alteration (<1 Mb) independent of alteration threshold; these regions were termed candidate true positives. In total 76% of all focal regions of alterations are identified in common between the SNP and Infinium arrays. Of those alterations showing a discrepancy between the Infinium and SNP arrays, approximately 25% are identified by the SNP array only (candidate false negative), while the remaining approximately 75% are identified by the Infinium array only (candidate false positive). The disparity in the call rates between the array platforms could be attributed to the differing array designs and the gene-centric nature of the Infinium arrays. When the analysis is limited to regions with sufficient probe coverage (minimum marker = 3) to call alterations in both arrays, over 79% of common alterations are detected. This resulted in an overall sensitivity of 0.71 and specificity of 0.83. To assess the performance of the Infinium array to detect CNAs with the same accuracy as SNP arrays, we plotted the binomial probability of an alteration being called a true positive versus alteration coverage at differing alteration thresholds (that is, gain, loss, amplification and deletion) across all 407 paired SNP Infinium array comparisons (Figure 6). This confirmed that the Infinium arrays show a good level of accuracy in detecting alterations at all levels of alteration across multiple studies (Table 2).

**Table 2:** Infinium sensitivity and specificity

	**Sensitivity**			
	**Deletion**	**Loss**	**Gain**	**Amplification**
Chondrosarcoma	0.83	0.91	0.89	0.69
GBM	0.97	0.85	0.87	0.75
Bladder cancer	0.62	0.81	0.79	0.67
Prostate cancer	0.6	0.81	0.85	0.63

The sensitivity and specificity of CNAs identified between SNP array and Infinium methylation arrays for four tumor types across a range of alteration types.

### Copy number alteration resolution

**Figure 7:**
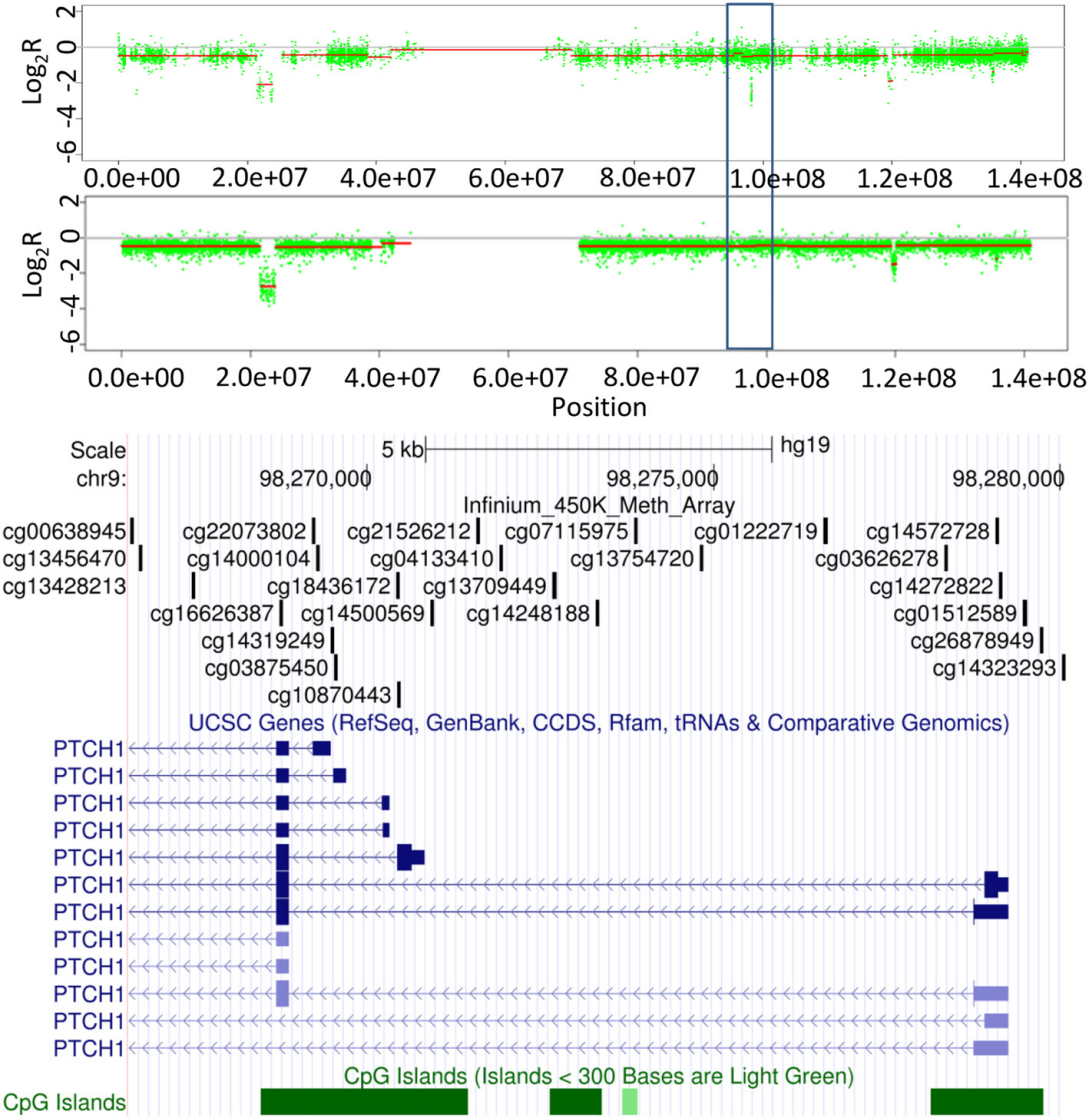
**Infinium unique *PTCH1 *deletion.** CN profiles for chromosome 9 for a single chondrosarcoma sample generated from Infinium HumanMethylation450 BeadChip (upper panel) and CytoSNP arrays (middle panel). The Infinium array-specific deletion of *PTCH1* is highlighted (blue box).The lower panel shows the genomic organization of the Infinium unique *PTCH1* region of deletion and the distribution of probes on the Infinium methylation, CytoSNP and SNP 6.0 arrays.

**Figure 8:**
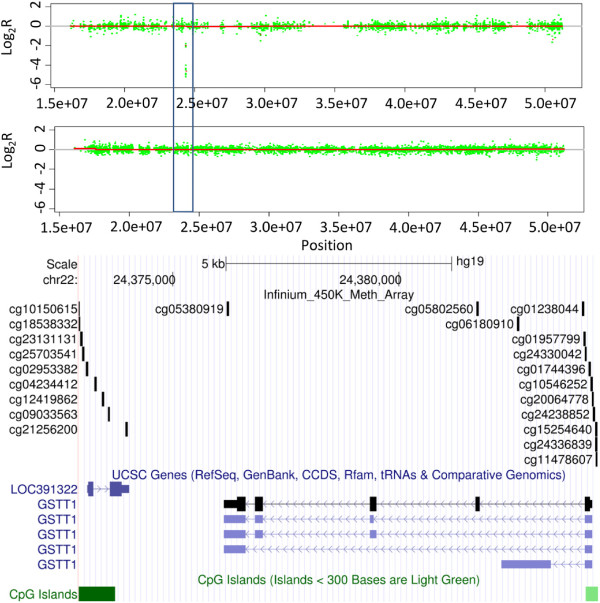
**Infinium unique *GSTT1 *deletion.** CN profiles for a proportion of chromosome 22 for a single chondrosarcoma sample generated from Infinium HumanMethylation450 BeadChip (upper panel) and CytoSNP arrays (middle panel). The Infinium array specific deletion of *GSTT1* is highlighted (blue box). The lower panel shows the genomic organization of the Infinium unique *GSTT1* region of deletion and the distribution of probes on the Infinium methylation, CytoSNP and SNP 6.0 arrays.

As highlighted above, Infinium arrays define a significant number of CNAs that are not present in the SNP array data (candidate false positives). We sought to determine whether these alterations are entirely down to array design or whether they were artifacts. On close inspection, most of these false positives (92%) appear to be regions devoid of sufficient probes to call a change on the SNP arrays. For example, Figure 7 shows LOH (loss of hetrozygosity) of the entire chromosome 9 by both SNP and Infinium arrays, along with the focal, potential homozygous deletion of a further four regions, including the loci encompassing the tumor suppressor gene *CDKN2A*. Three of the four homozygous deletions are identified by both array types, apart from an approximately 10 kb region (Figure 7) not detected by the CytoSNP array. This region (log_2_R = -2.7) contains 24 probes on the Infinium arrays and appears to span approximately 34 kb (first 3 exons) and 1.2 kb upstream of *PTCH1* only (9 probes in the remaining 44 kb of *PTCH1* showed heterozygous loss only, log_2_R = -0.36, similar to the remainder of Chr9). However, this region is represented by only a single probe on the CytoSNP array (nearest neighbors 5′ = 7.34 kb and 3′ = 6.99 kb). Quantitative PCR validation confirmed the heterozygous deletion of this region in *PTCH1* (Figure S4 in Additional file 1). Similarly, Figure 4 shows the homozygous deletion of a small region centered on *GSTT1*; homozygous deletion of this gene has been associated with increased susceptibility to many different cancer types, including prostate cancer, renal cancers and osteosarcoma [32-35]. The Infinium data indicate this deletion spans approximately 12 kb and contains *GSTT1* and a small proportion of the neighboring *LOC391322* only (Figure 8). This region also contains a single probe from the Affymetrix SNP6.0 array and would be undetectable by the Illumina CytoSNP arrays (Figure 8). Quantitative PCR validated the homozygous deletion of *GSTT1* (Figure S4 in Additional file 1). Although we have not mapped the full extent of these deletions, these data highlight the potential utility of these arrays to identify novel small alterations that are not detectable with existing SNP array platforms.

We further validated CNAs identified by the Infinium arrays with the targeted exome-sequencing of key cancer genes [36]. This analysis revealed greater than 90% concordance between alterations identified by Infinium CNA profiling and targeted exome sequencing (Figure S5 in Additional file 1). Of overlapping loci, 45 alterations were identified from Infinium CNA profiling with a false positive rate of 8%, and a similar false negative rate (8.8%) [36], further highlighting that the Infinium arrays provide a reliable, robust and cost-effective method of identifying CNAs in human cancers.

## Conclusion

There is increasing interest in the integration of genomic and epigenomic data from the same DNA specimen in order to provide greater insight into disease processes. It is particularly intriguing to integrate genomic CN and DNA methylation data, which may allow the identification of synergistic mechanisms for the inactivation of tumor suppressor genes or the activation of oncogenic pathways [3]. However, the integration and ultimately the interpretation of these integrated datasets are both costly and challenging if carried out separately.

Here we sought to evaluate whether the Infinium HumanMethylation450 BeadChip could be utilized to determine CNAs as well as epigenetic alterations. Initially, we sought to confirm that the methylation state inferred by the Infinium HumanMethylation450 BeadChip was not biased by altered CN state. We show there is little bias when comparing normal (two copies) to heterozygous loss (one copy) or single copy gain (three copies). However, there does appear to be a correlation at loci of complete genomic loss, potential homozygous deletion (more than one copy) and amplification (more than four copies). Association of methylation and CNA state with homozygous loss is unsurprising and has little impact on methylation analysis *per se* as these loci are generally removed from methylation analysis due to signal intensities indistinguishable from background (low detection *P*-value). However, it may represent a confounding factor effect when comparing methylation in samples with and without CNA. For example, a tumor suppressor deleted in a proportion of samples may be hypermethylated in others, but in many Infinium methylation array analysis pipelines this information will be lost due to the removal of missing data. This highlights the importance of integrated analysis using both CNA and methylation data. The strong negative association between methylation state and regions of high level amplification was less anticipated, and appears to be a result of the genomic distribution of probes as opposed to inherent biases of the arrays. As most probes in regions of amplification fall within CpG islands, which are predominately unmethylated, these therefore contribute to the apparent loss of methylation in regions of amplification.

Our primary objective was to assess whether the Infinium HumanMethylation450 BeadChip could be used to accurately assess CNAs to the same degree of reliability and sensitivity as standard SNP array platforms, such as the Affymetrix 6.0 SNP or Illumina CytoSNP arrays. Specifically, we compared Infinium CNA profiles from samples with matched SNP array data. Using the same algorithm for all array types, we show that approximately 85% of all alterations were identified in both SNP and Infinium arrays (when regions contain sufficient overlapping probes). Interestingly, we see a reduced concordance when assessing smaller alterations, with a high number of false positive alterations identified by the Infinium arrays compared to SNP platforms. The majority of these appear to be results of differences in array design and the gene-centric design bias of the Infinium arrays. Unlike the standard SNP array design, with probes roughly evenly distributed throughout the genome, the Infinium arrays are very much gene-centric in their design, with 95% of probes within 2 kb of 95% of the known genes and, on average, >9 probes per gene. Therefore, although the Infinium arrays may lack the resolution of SNP arrays to detect alterations in large intergenic regions or gene desert regions, they provide high resolution coverage of the majority of coding loci. This allows for the identification of discreet alterations of individual genes, which would not be detected by standard SNP arrays. Similarly, with over 94% of CpG islands represented, these arrays may also allow the identification of small alterations within regulatory regions, potentially revealing novel mechanisms of gene disregulation. Therefore, the gene-centric/biased design of the Infinium array has a greater potential to identify driver CNAs involved in tumorigenic processes.

Furthermore, as the same loci can be interrogated for both methylation and CN in the same DNA sample, the analysis potentially allows easier integration of epigenetic and genomic data. The integration of methylation and CN data can provide fascinating insights into the underlying biology of malignant processes where the challenge is to identify driver from passenger alterations [3]. For instance, a change in genomic content (that is, single copy gain or loss) does not have to correlate with a linear change in methylation; in fact, it is those genes that show an inverse correlation between CNA and methylation that may be most important. For example, tumor suppressor genes that undergo a ‘double hit’ - that is, heterozygous loss and hypermethylation - or oncogenes in a region of gain that are hypomethylated compared with neighboring genes may represent those genes most likely to be differentially expressed and consequently drivers of tumorigenic processes. Hence, through utilizing the Infinium arrays for both epigenetic and CN analysis, it may be possible to more accurately distinguish between genes that drive the selection of a malignant phenotype from those that are passengers within an amplified or deleted region.

Finally, it can be difficult to compare CNA data across different high-density array platforms, particularly given differing designs, and even the comparison of the same data with differing algorithms can lead to varying results [37-39]. Even given these caveats, these data show the utility of using the Infinium HumanMethylation450 BeadChips to define CNAs in human cancers. We show that the Infinium Arrays are as robust and sensitive as current high density SNP arrays for the detection of CNAs and appear highly applicable for providing estimates of CN as well as a measure of methylation state. Furthermore, we highlight that the gene centric design of the arrays may be beneficial, in allowing the identification of alterations containing single genes or just regulatory regions, which may aid in our understanding of the complex genomic and epigenomic interactions driving the development and progression of a malignant phenotype.

## Materials and methods

### Study population

DNA from 11 chondrosarcoma specimens were subjected to profiling on Infinium HumanMethylation450 BeadChip and HumanCytoSNP-12 BeadChip (GSE40853) [40]. The material was obtained from the RNOH Musculoskeletal Biobank, with approval provided by the Cambridgeshire 1 Research Ethics Committee (reference number 09/H0304/78).

Infinium methylation data with matched targeted exome-seq data were generated from 44 formalin-fixed paraffin wax-embedded (FFPE) head and neck squamous cell carcinoma (HNSCC) samples [41] (GSE38271, SRP034519). Ethical approval for these samples was granted by the UCL/UCLH Ethics Committee (reference number 04/Q0505/59).

Finally, matched Infinium array and Affymetrix SNP6.0 array data were downloaded from TCGA DataPortal for 74 GBM samples [42] and for 178 prostate cancer samples [43].

### Genome-wide methylation profiling

For chondrosarcoma and HNSCC, 1 μg of DNA from fresh frozen tissue and 2 μg from FFPE tissues [41] were bisulfite converted using the EZ DNA Methylation kit (Zymo Research Corp. Irvine, CA, USA) according to the manufacturer’s instructions, with the exception of FFPE samples, which were bisulfite converted using a modified protocol [44]. Bisulphite converted samples were processed and hybridized to the Infinium HumanMethylation450 BeadChip according to the manufacturer’s recommendations. Subsequent data were processed and beta values computed using the methylation module of the GenomeStudio software (version 1.9.0; Illumina). Briefly, each CpG locus interrogated is represented by signals corresponding to both the methylated (M) and unmethylated (U) alleles, respectively. The beta value represents the ratio of the intensity of the methylated bead type to the combined locus intensity: *β* = max(M, 0)/(max(M,0) + max(U,0) + 100) and reflects the methylation status of a specific CpG site.

### CytoSNP

DNA (300 ng) from 11 chondrosarcoma specimens and one normal reference DNA sample were processed and hybridized to the HumanCytoSNP-12 BeadChip according to the manufacturer’s instructions. Subsequent data were processed and R values computed using the genotyping module of the GenomeStudio software (version 1.9.0; Illumina). Further analysis and identification of CNAs was carried out in R (version 2.15.0) [45].

### Identification of copy number alterations

CNA data were generated from un-normalized signal intensities. Signal intensities were extracted for each sample using GenomeStudio. Probe intensities were subsequently subjected to GC content normalization, carried out using cyclic loess and log2 ratios, generated to averaged normal reference samples [25]. Circular binary segmentation, from the R package DNAcopy, was then performed to define chromosomal segments with differing CN states, with the following settings: alpha = 0.001, undo.splits = ‘sdundo’, min.wdith = 3 [31]. Thresholds for the identification of single copy CNAs were derived from the difference in log ratio between normal reference DNA from male and female samples (log2 ± 0.33), denoting a single copy change in the X chromosome; high-level amplifications and homozygous deletions were defined incrementally from this threshold. The level of noise was determined from the median deviance of neighboring probes. Probes that show a high degree of variability, such as the highly polymorphic major histocompatibility (MHC) region on the short arm of chromosome 6, were removed from subsequent analysis.

This method for identifying CNAs from the Infinium methylation arrays is incorporated in the ChAMP Bioconductor package [46,47], an Infinium HumanMethylation450K array integrated analysis pipeline that allows quality control, normalization, calling of differentially methylated regions and methylation variable positions along with detection of CNAs [47].

Copy number alterations from reference CytoSNP arrays were generated with DNAcopy (chondrosarcomas) as above from normalized R values. We analyzed publicly available GBM Affymetrix SNP6.0 segmented data to identify CNAs. Thresholds derived from the difference between sex chromosomes in male and female patients was used to identify single CN gains and homozygous deletions. Amplifications and homozygous deletions were assessed using incremental thresholds.

### Correlation between Infinium and SNP array-defined CNAs

Regression analysis was used to determine the association between signal intensities and CNAs from the Infinium HumanMethylation450 BeadChip and CNA status defined from SNP arrays (Affymetrix SNP6.0 or Illumina CytoSNP). This was carried out in R using Bioconductor packages glm or gam. The Bioconductor packages and iRanges [48] were used to define overlapping regions between Infinium and SNP array CNA data from all 407 paired samples.

Binomial probabilities of true positive detection were calculated across all 407 samples at any given CNA alteration threshold (deletion, loss, gain or amplification). We define true positive binomial probabilities first by defining true positive counts. The true positive count is defined as the number of overlapping regions between paired samples on any two platforms at any given alteration threshold and alteration size. A binomial test was used to convert true positive counts to binomial probabilities with 95% confidence intervals for each sample comparison.

Sensitivity was defined by the number of true positives over the total number of alterations (true positives plus false negatives) detected by the Infinium array at any given alteration threshold. Specificity was determined by the Infinium false positive call rate (that is, an Infinium CNA identified in a region of no change defied by the SNP array). True negatives were defined as overlapping genomic regions without alteration on both platforms, compared to the number of Infinium false positives plus true negatives. Only windows with more than three probes in both platforms were assessed.

### Targeted exome sequence analysis

Matched tumor and germline DNA from 44 FFPE HNSCC samples were subjected to targeted exome capture and next-generation sequencing [36,41]. Briefly, exome sequencing was carried out using a custom SureSelect capture kit, representing 3,230 exons in 182 cancer-related genes plus 37 introns from 14 genes often rearranged in cancer. Paired-end sequencing was performed using the HiSeq2000 (Illumina). Reads were subsequently mapped to the reference human genome (hg19) using the BWA aligner and processed using SAMtools [49], Picard [50] and the Genome Analysis Toolkit (GATK) [51]. CNAs were detected by comparing targeted genomic DNA sequence coverage with a process-matched normal control sample. Genomic rearrangements were detected by clustering chimeric reads mapping to targeted introns [36,47].

### Quantitative PCR validation of alterations

Deletions of *PTCH1* (chromosome 9) and *GSTT1* (chromosome 22) were validated in triplicate biological replicates using SYBR-Green quantitative PCR. Loss of these regions was determined relative to the control gene *ACTB* (chromosome 7), a universal housekeeping gene.

## Abbreviations

CN: copy number; CNA: copy number alteration; GBM: glioblastoma multiforme; HNSCC: head and neck squamous cell carcinoma; ICGC: International Cancer Genome Consortium; SNP: single-nucleotide polymorphism; TCGA: The Cancer Genome Atlas.

## Competing interests

The authors declare that they have no competing interest.

## Authors’ contributions

AF conceived and developed the method for identifying CNAs from Infinium arrays and wrote the manuscript. TJM incorporated the method in the ChAMP pipeline. GAW and AET provided bioinformatics and statistical support. ML, PG, TF, and AMF provided samples and data. SB and JDK supervised the study and CT, SB and JDK contributed to writing the manuscript. All authors have read and approved the final manuscript.

## Supplementary Material

Additional file 1: Figures S1 to S5 **Figure S1.** Distribution of beta values for promoter associated and non-associated CpGs. **(A)** Boxplot of beta values for CpGs in non-CpG island associated promoters and CpG island associated promoters for regions of genomic amplification. **(B)** Regions of genomic deletion, **(C)** Regions of genomic loss, **(D)** CNA neutral regions and **(E)** Regions of single copy gain. Click here for file
