# Supplementary material for: Using high-density DNA methylation arrays to profile copy number alterations
Source: Genome Biol. 2014 Feb 3;15(2):R30. doi: 10.1186/gb-2014-15-2-r30 (PMC4054098; doi:10.1186/gb-2014-15-2-r30)

Supplementary Figure 1. Boxplot of probe distribution in regions of A) amplification, B)Deletion, C) Loss, D)Normal, E)Gain.

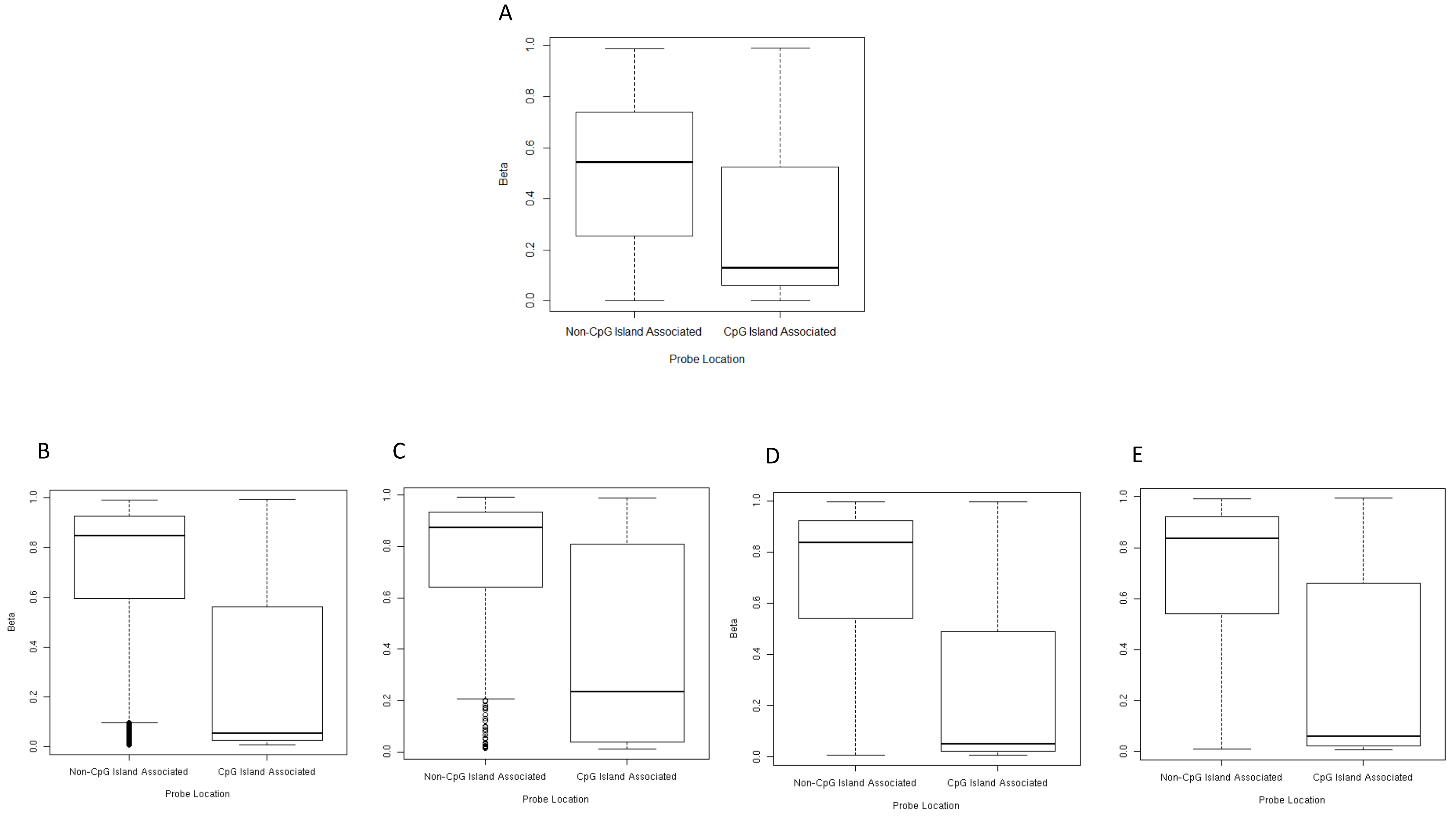

Supplementary Figure 2. Batch correction of replicate samples. A) Violin plot of Infinium array intensities for replicate samples across multiple batches in institutes. B) Violin plot of ComBat batch corrected intensities , C)Scatter plot of replicate samples from the same batch and institute, D) scatter plot of replicate sample across batches from the same institute, E) Scatter plot of replicate samples a from different sites, f) Scatter plot of batch corrected intensities from different sites.

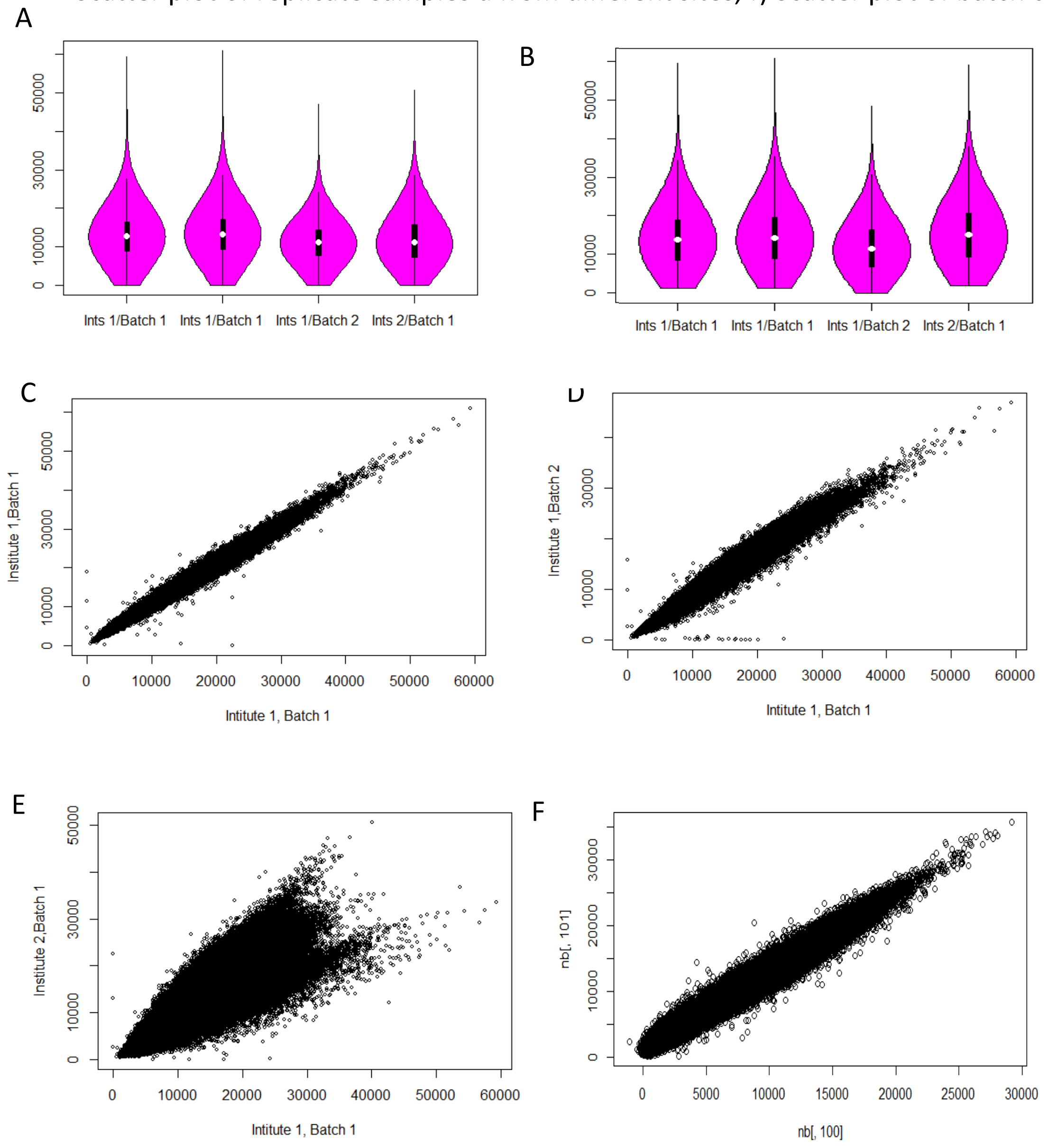

Supplementary Figure 3. CN prolifes comparing Infinium probe type. Copy number profiles generated using DNAcopy from A) Type I, B) TypeII and C) combined.

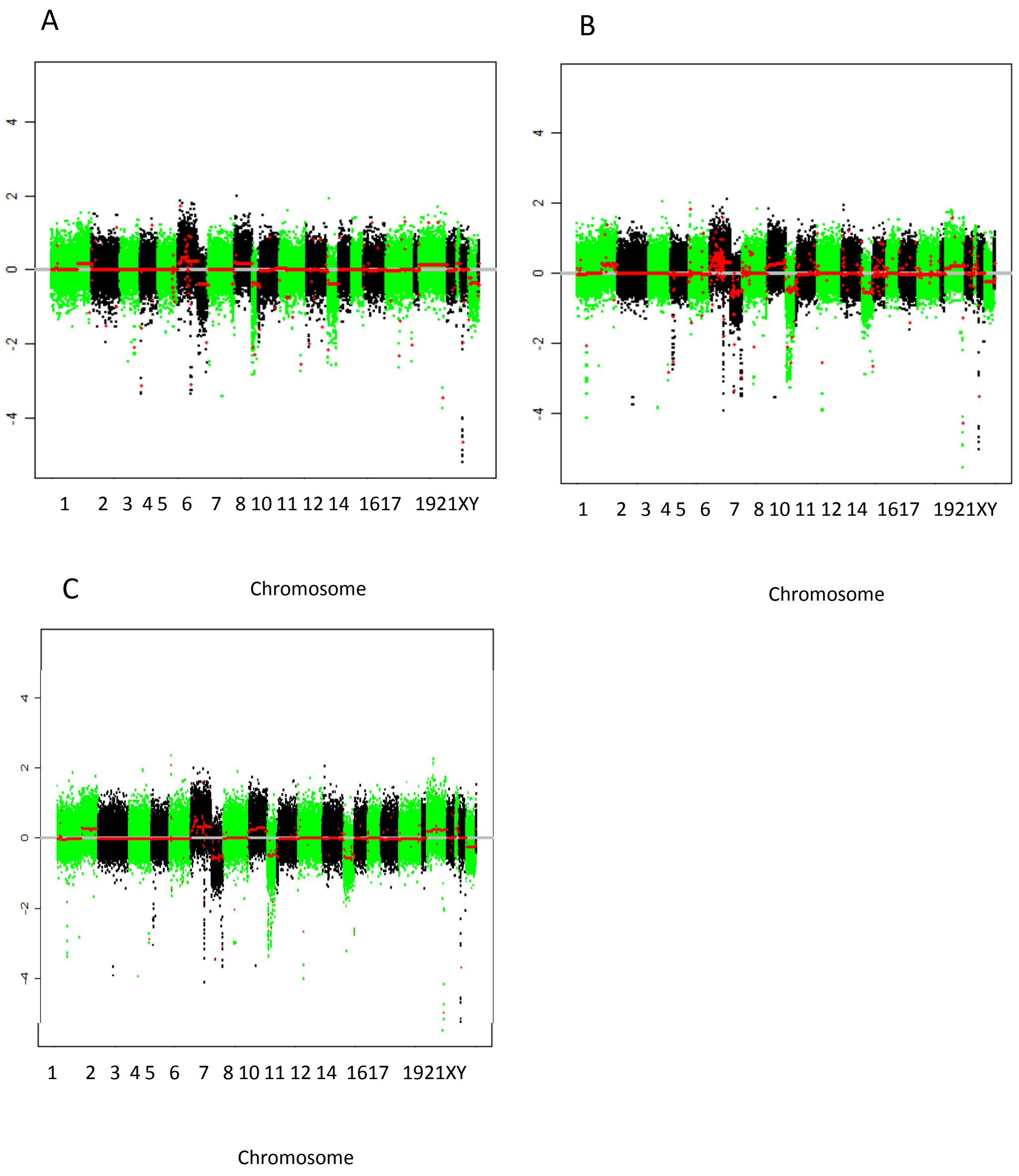

Supplementary Figure 4. Q-PCR validation of Infinium specific deletions in a deleted and non deleted sample. A) 9q22 deletion of *PTCH1*, compared to a reference of B-Actin and an internal Chr 9p region. B) Q-PCR validation of *GSTT1* deletion, compared to a reference of B-Actin. Reactions were performed in triplicate, Error bars are shown in red.

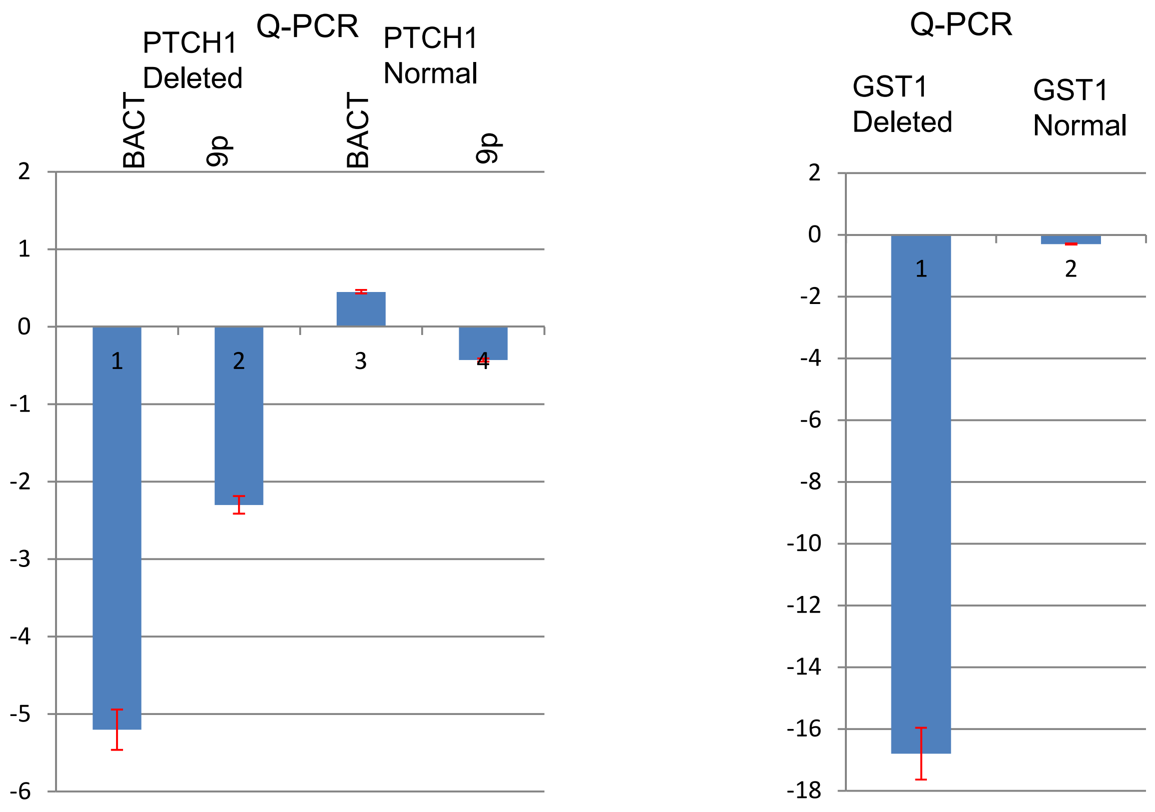

Supplementary Figure 5. Comparison of CNAs identified by Infinium and Exome-Seq, 42 of 44 (95%) copy number alterations detected by sequencing were confirmed (green: confirmed, pink: not confirmed); Lechner et al., 2012 (submitted)

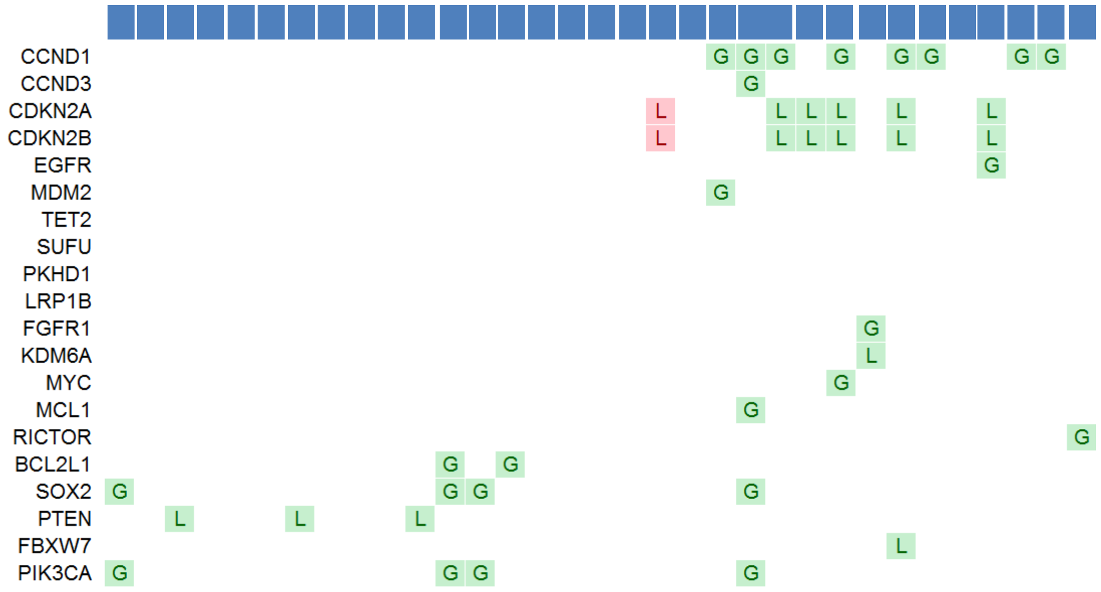

Supplement: Additional file 1: Figures S1 to S5 — Figure S1. Distribution of beta values for promoter associated and non-associated CpGs. (A) Boxplot of beta values for CpGs in non-CpG island associated promoters and CpG island associated promoters for regions of genomic amplification. (B) Regions of genomic deletion, (C) Regions of genomic loss, (D) CNA neutral regions and (E) Regions of single copy gain. [file gb-2014-15-2-r30-S1.pdf]
